# ***The President’s Physician: An African Play***: **Emmanuel Babatunde Omobowale, 2004, All Saints’ Publishers (Ibadan, 978-978-37727-3-1, 77 pp.)**

**DOI:** 10.1007/s11673-017-9811-z

**Published:** 2017-10-11

**Authors:** Joseph Ajagunmolu Mayaki

**Affiliations:** Edo University, Iyamho, Edo State Nigeria

**Keywords:** Literature and medicine, Biomedical ethics, Utilitarianism, *The President’s Physician*, Emmanuel Babatunde Omobowale

## Abstract

This review examines issues relating to biomedical ethics and literature in the African drama *The President’s Physician* by Emmanuel Babatunde Omobowale. The play investigates the psychological dilemma of Doctor Bituki Warunga, a personal physician to General Kalunga Ntibantunganyah who brutally and inhumanely rules Wavaria, a fictional African country. The doctor is faced with deciding to uphold the ethics of his profession versus terminating the tyrant’s life to set the nation free. The play aims to help budding medical doctors rightly inculcate the principles of medical ethics—autonomy, beneficence, competence, and power—by providing a fictional platform to investigate difficult issues that can arise in clinical practice. The play highlights Warunga’s complex dilemma as he struggles to uphold the Hippocratic Oath and at the same time satisfy his conscience towards his contribution to his country’s freedom. This review explores the difficulties in decision-making when professional duties not only clash with personal preferences but also with the well-being of an entire nation. This discussion is done alongside the ethical concept of utilitarianism and also highlights significant literary concepts such as satire, symbolism, intertextuality, utopian aesthetics, and authorial vision as conveyed in the text.

Literature and medicine is a relatively new area of intellectual enquiry, most especially in Africa. This discipline intermarries two (what some may claim as) diametrically opposed fields of study. However, it is interesting to note that the sociological approach to literary evaluation has been critiqued for making literature a study of other disciplines (Barry 1995; Bamidele 2000); yet because literature cannot really be separated from society, it has not ceased to enquire into human problems or in a way suggest or proffer solutions to tackle them. This underscores its functional dimension. Literature and medicine, therefore, can be seen as a unique field that came about partly due to the compelling utilitarian relevance of literature (Charon 2000; Skelton, Macleod, and Thomas 2000). For medicine to faithfully serve humanity, medical doctors have to understand the connection between the human mind and body (Omobowale 2003) and not restrict themselves to the study of anatomy, biochemistry, and physiology alone. The human mind and body are two inseparable entities, and one cannot successfully and entirely function without the other (Windish et al. 2005). R.S. Downie in “Literature and Medicine” (1991) explains important points of convergence between the two. He reiterates the fact that among the important world literary writers are medical doctors, such as Anton Chekhov, the Russian playwright and author of short stories. Chekhov takes pleasure in saying that medicine was his first wife and literature his mistress (cited in Downie 1991, 93), and he examines issues that are perhaps best investigated by a physician–writer. This is partly why Downie opines:

Clearly, literature is enriched by the insights of those who are on the inside of medical situations, and the medical profession must surely be grateful that some of their members have the literary skills to convey this special insight to the public (Downie 1991, 93).

Downie sees the handling of medical doctors by non-physician writers in fictional works as a serious point of contention. He argues that medical doctors are often portrayed as rapacious and self-centred, which is not truly what really obtains. But physician–writers can help give a more honest portrait of doctors in a literary piece. Additionally and importantly, Downie also underscores that literature can enhance a medical doctor’s understanding of the whole person in the doctor–patient relationship. These and other medical related issues are explored in Emmanuel Babatunde Omobowale’s play, *The President’s Physician*.

## Emmanuel Babatunde Omobowale: Brief Biodata

Emmanuel Babatunde Omobowale is a professor in and the chair of the Department of English at the University of Ibadan, where he teaches courses in bioethics, creative writing, African and black diaspora literature, and literature and medicine. He has an M.A. degree in bioethics from Case Western Reserve University in the United States as well as a B.A., M.A., and Ph.D. in English from the University of Ibadan in Nigeria. He is also Nigeria’s first professor of literature and the medical humanities. He is a contemporary African creative writer, and his creative works include the novella *The Eagles Must Fly* (1992), a collection of short stories entitled *The Melting Pot* (1993), and the novel *Seasons of Rage* (1997). Omobowale hails from a Christian home. His father is the founder and senior pastor of All Saints Evangelical Church in Ibadan, where Omobowale is also an officiating minister. His pacifist Christian beliefs are creatively fused into his revolutionary literary works. The plots of his stories are structured such that the oppressed gets justice without necessarily resorting to violent confrontations (Veatch 2012). Such is also the case in *The President’s Physician*.

## *The President’s Physician*: An Overview

*The President’s Physician* is an African play that investigates the psychological dilemma of Doctor Bituki Warunga, a personal physician to General Kalunga Ntibantunganyah who brutally and inhumanely rules Wavaria, a fictional African country. The doctor is faced with deciding to uphold the ethics of his profession versus terminating the tyrant’s life to set the nation free. The play aims to teach budding medical doctors the principles of medical ethics: autonomy, beneficence, competence, and power (Clark 2004).

The play has five movements with irregular lengths. General Ntibantunganyah, former aide-de-camp to the prior leader Magundare, comes into power by overthrowing Magundare’s tyrannical regime, though only to continue the oppressive rule. He kills Major Akubor for opposing him, rendering Akubor’s young, pregnant wife, Rachael, a widow. He also kills his daughter’s fiancé. Her reaction to this offers insight into Ntibantunganyah as well as commentary on the freedom and burdens of truth:

Oh! Yes you know what I mean, father. You sent your boys to kill Takoh, on account of his father’s opposition of your government. He was the love of my life. We planned to get married, but you killed him. You never wanted me to associate with the man that I loved, because he was the son of your enemy. Now, you pretend not to know why I am sick. You asked your bodyguards to give me cocaine so that I will not be able to think coherently and constructively. Now, I know the truth and the truth has enslaved me (Omobowale 2004, 59).

Ntibantunganyah is far from a virtuous leader or man. He impregnates numerous young girls and forces them to undergo abortions; embezzles from public funds; and forcefully takes the sacred land of the rural community called Akampehi for a medical research project and obtains blood samples from their pregnant women and children without permission. Threats made by Ntibantunganyah’s wife, Akwabah, to Kajolah, chief of the Akampehi, shed light on this:

Tomorrow, trucks from the Ministry of Works and Housing will be off-loading building materials at the site. The Ministry of Justice has already prepared a decree to be signed by my husband appropriating the land to me. The land is mine and the research shall be conducted on those women and children. […] But I warn you, if any one tries to stop Akwabah Ntibantunganyah, not even Nuranda [meaning God] will be able to save you from my wrath. Think about it (Omobowale 2004, 49, 50).

As Omobowale makes clear in the early movements of the play, Ntibantunganyah’s actions have been evil, so does he deserve to be spared by a medical doctor who could easily and quietly snuff out his life and save the Wavarians? This is an ethical dilemma for a doctor.

By faithfully administering drugs to his patient and nurturing his life, Doctor Warunga in a way helps to sustain Ntibantunganyah’s reign of terror, elongating the suffering of the entire nation. In serving Ntibantunganyah, Warunga also violates other people’s rights to please the president. At the president’s recommendation, he confines Eve, the president’s daughter who opposes her father’s methods, to a psychiatric institution. As the chief medical director, he does not question the illegal abortions carried out at the hospital. Even though the president’s actions and desires tarnish Warunga’s public image and reputation, he resists subtle suggestions from his own father, also a medical doctor, to annihilate the leader. But Warunga vacillates in his dilemma. In a dream, Warunga confronts the president and almost kills him; however, he is grateful to wake up, but then later rejoices at the death of the president and the president’s accomplices.

Despite his service to the president, Warunga really wishes him dead. From a psychoanalytic perspective, it would not be incorrect to conclude that this is Warunga’s inner desire, maybe even part of his true personality. Is Warunga a nice person? A nice physician? Is he a faithful and objectively dutiful medical practitioner? On the other hand, does he not owe the oppressed Wavarians any duty? How patriotic can he be said to be? These are the ethical questions explored in the play.

Warunga’s attitude and actions towards the condition of the Wavarians are disconsonant with Major Akubor’s, who appears as a sacrificial idealist in the play and one who believes in justice. Even Warunga himself talks of Akubor thusly:

He rejected oppression and recommended fair play and trust as a basis for any meaningful, positive relationship between the rulers and the ruled. He despised evil and anything that could tarnish the image of the military. He was an upright soldier and to him, Ntibantunganyah’s desire to rule longer than expected was a despicable act of greed. He was a rare breed and he proved to all and sundry that he was an officer and a gentleman and an ideal example of a responsible individual. But ours is not an ideal society. Whoever seeks the freedom of citizenry will always be regarded as a snag in the wheel of progress (Omobowale 2004, 24).

Akubor loves truth and what is right more than his life or comforts. Between him and the supposedly nice physician Warunga, who is a hero? Who should be the hero of this play?

## Ethics and Biomedical Ethics in the Play

Ethics is viewed as a system of moral principles that governs the appropriate conduct for a person or group. It has to do with the application of values and moral rules to human activities. Biomedical ethics, on the other hand, is a subset of ethics and uses the principles of ethics and decision-making to solve actual or anticipated problems in medicine. For doctors, bioethics often is tied to the Hippocratic Oath, which guides physicians to maintain strict confidentiality in treating patients and against causing harm, performing abortions, and prescribing deadly drugs (American Medical Association and New York Academy of Medicine 1848).

It is important to note that biomedical ethics can sometimes conflict with law, which largely regulates the practice of medicine. It is true that good ethics often makes good law, whereas good law does not necessarily make good ethics. Take, for instance, Ntibantunganyah: Warunga’s patient is dangerous to the Wavarians. He keeps a whole nation in bondage. He transgresses their civil rights. Is he not an enemy of the state? Is he not a bane to the country’s progress and development? Why should the doctor continue to promote his health in obedience to the Hippocratic Oath upheld by medical ethics? In Ntibantunganyah’s own words:

I am selfish and greedy and I know that I cannot stop embezzling government funds or stop killing real and imagined enemies. And if I cannot help myself, how can I help others? My life has always threaded the path of brutality. As a leader, there is only one thing I know, the use of force, I seek people’s cooperation by force. [...] No one, not even a dying patient or a woman in labour pain, is more important than Kalunga Ntibantunganyah (Omobowale 2004, 29, 46).

Is such a brutal man worthy of being preserved by a doctor? Ought he not to be killed? If this man were an ordinary citizen in police custody, he would be sentenced to die by hanging or firing squad. The doctor seems to prioritize keeping the president alive over an orderly humane society. Is there justice in Warunga’s handling of Ntibantunganyah’s case? It isn’t an easy question to answer bearing in mind that a whole nation is trapped because this man is still alive! It is proper to commend Warunga for his faithfulness to the ethics of his profession; he cares for the brute despite the inconveniences and dissatisfaction it gives him, as he expresses:

I am obliged to live in this accursed house for a single reason, to provide His Excellency medical attention to avert his untimely death, which can be caused by one of the many deadly ailments plaguing his body. He believes that I am the only well trained medical doctor in Wavaria, who can keep him from joining his forebears in hell, where I believe he truly belongs. [...] Ntibantunganyah is a hypochondriac who suffers from a mixture of real and imagined diseases, which has necessitated my ubiquitous presence by his side every day and every night. He would have died a long time ago if it were not for my meticulous attention to his medical needs (Omobowale 2004, 5, 6).

Utilitarianism, an ethical doctrine that promotes the greatest good, states via its greatest happiness principle that the criterion of the virtuousness of an action is to maximize pleasure and minimize pain for all. Can this be applied to the case at hand, if the doctor refuses to treat or even kill his neurotic president–patient in order to save the nation from the tyrant’s oppressive grip? Or does this pervert utilitarianism by falsely justifying murder as the right course of action? Doctor Warunga sustains Ntibantunganyah to the detriment of the nation. Is there a gap in his understanding? This is critically considered in the interaction between Chief Kajolah, the ruler of the Akampehi community, and Warunga, when Ntibantunganyah’s wife, Akwabah, seeks to take blood samples from the women of the community—revealing more details about Warunga’s personality and understanding of and attitude towards biomedical ethics.

**Kajolah:** […] One important question, which I want to ask you, is this: have you sought the permission of the women and their husbands before you begin to do your research? You cannot just go into their compounds and begin to ask them questions. A few years ago, some scientists came here from the United States to conduct research on the guinea worm epidemic, which was ravaging Acampehi at the time.^1^ They got permission from me. My chiefs as well as the people were informed before they started asking any questions about their ailments from them. They were also financially compensated on the days when they did not go to their farms because they had appointments with the scientists. Do you and Akwabah intend to do this?

**Bituki [Warunga]:** Your Highness, the First Lady does not intend to seek anybody’s consent before embarking on the research. Soldiers and policemen will be used to enforce her will and ensure the participation of the people. I cannot advise her on this. I fear for my life (Omobowale 2004, 50, 51).

As can be seen in this exchange, Warunga upholds bioethical tenets when the president is his patient but at the president’s instruction violates these same rules in dealing with others. What kind of a physician is Warunga? In his position as the president’s physician, should he be a model? He might be a professionally adequate medical doctor, but should he also be a virtuous one, and if so, what would this look like in his role? Is he truly convinced that a patient’s autonomy should be respected? This becomes circumspect when he disregards the autonomy of the Akampehi women as research subjects. And with regard to “doing no harm,” how does his dream of confronting and attempting to kill the president fit within his consciousness and unconsciousness? He eventually rejoices at Ntibantunganyah’s death:

It was the biggest New Year eve’s fireworks ever witnessed in Wavaria. I have never rejoiced over the demise of anyone before but I felt some relief on that particular night. After 20 years of enjoying absolute power in Wavaria, Ntibantunganyah had taken himself and the cream of his army with him into the depths of hell. I felt extremely happy. Two weeks after his death, I performed an autopsy on his mangled body. It was my last official duty as the personal physician to the president (Omobowale 2004, 69).

Considering the intricate web Warunga finds himself in, he can be judged to have been professionally fair to Ntibantunganyah, his patient. However, he does not uphold his professional principles in all cases.

## Literary Aesthetics in the Play

The play is enriched by a number of literary aesthetics and techniques. This is essential for any play or work of prose or poetry to qualify as “Literature” with an upper-case “L.”

### Satire

The practice of military dictators seizing power by force and ruling the people oppressively is a prevalent issue in Africa. Charles Taylor of Liberia, Idi Amin of Uganda, and Abacha of Nigeria are a few examples. Likewise, highly placed government officials and politicians embezzle funds to the detriment of the masses. Omobowale has reflected these social realities of mis-governance and oppression in his play as a caricature of what truly obtains with the hopes of correcting them. There are also officials like Warunga who serve brutish leaders and struggle with defining what should be the extent of their loyalty for the good of the entire masses. Ntibantunganyah has been sarcastically characterized, a maniac who insists on being the president despite his evident mental and moral lapses. The idea of African (and other) leaders wanting to hold on to power for life and their view that this is their birthright is also ridiculed by the inglorious end of Ntibantunganyah. The monopoly of political power by officials is common in Africa. A recent case is that of Yahya Jammeh of The Gambia, who initially refused to step down as president after losing his country’s December 2016 election. He eventually stepped down at the intervention of a West African military alliance. This is a man who came into power through a bloodless military coup. Having ruled for twenty-two years, he still desires to be the sole ruler of his country against the wishes of the majority. It is worthy to commend the ethical decisions of certain Jammeh officials, including his vice president, who resigned their positions before Jammeh eventually stepped down. By these resignations, they declared their disapproval of Jammeh’s selfish ambition and contempt for the wishes of the people and the law of their country. This is something Warunga could have done as the tyrant’s physician.

### Symbolism

Characterization in this play has been symbolically employed. The characters of Warunga and Akubor serve as substitutes for the ideologies of pacifism and Marxism, respectively. Warunga represents the belief that violence, war, and the taking of lives are unacceptable means of resolving differences. This can possibly account for why Warunga says—in his talk with Chief Kajolah as he enforces the unethical research on the Akampehi people (see above)—“but if you believe in the Supreme Being like I do, please pray that we overcome the menace of Ntibantunganyah” (Omobowale 2004, 51). Warunga speaks as a believer in the Christian ethics of pacifism and divine retribution. He does not think he has the power to decide the tyrant’s fate but instead resorts to prayers.

The Marxist Akubor, however, believes in revolution by every possible means, even if it will mean his premature death. He does not believe in maintaining the status quo. He questions Ntibantunganyah’s erring authority and proudly gives his life for this course. His conversation with his colleague, Colonel Agunyah, at the sixtieth birthday ceremony of Ntibantunganyah sheds light on this:

The President should go to hell. As soldiers, we serve as guardians of the people and we must not let them down. We must perform our constitutional role with dignity. Military rule seems a never-ending process in Africa; why must that be? [...] Look at what happened the other night. About a dozen innocent soldiers who voted against Ntibantunganyah’s decision to continue to rule were arrested and incarcerated. Six of them were executed last week, killed on the orders of the President to celebrate the twentieth anniversary of his ascension to power. Can you just imagine that? It is an unnecessary shedding of human blood. Now today, as he marks his sixtieth birthday anniversary, six more people have been murdered in cold blood. This regime personifies evil, Agunyah, and I feel very sad. [...] I have already decided on what I want to do. It is too late to go back now. My mind is made up. I will start by publishing an article that will expose Ntibantunganyah’s excesses in the national newspapers (Omobowale 2004, 12, 18).

### Intertextuality

Intertextuality is a deconstructionist term coined by Julia Kristeva (cited in Baldick 2001, 128) to designate the various relationships that a text has with others. The attitude of Warunga reminds readers and viewers of the African American historical figure Booker T. Washington, a notable educator who discouraged violence in the fight to oppose Jim Crow disenfranchisement in the American South following the abolition of slavery. Some feel he was too apologetic and timid in his approach (Du Bois 1903). Conversely, Akubor resembles W.E.B. Du Bois, the sociologist and social reformer and the first African American in the United States to earn a Ph.D. He, like Akubor, had a radical, confrontational approach in his fight against oppression and segregation (Du Bois 1903). Akubor can also be likened to Ngugi and Mugo’s (1976) Dedan Kimathi in *The Trial of Dedan Kimathi*, who confronted the colonizers in his struggle for Kenya’s liberation (Dasylva 2004).

### Utopian Aesthetics

Sir Thomas More coined this term in 1516 to mean a good place that exists in no place (cited in Baldick 2001, 269). It is an ideal but non-existent political and social way of life. The way Warunga is portrayed is overly idealistic. Most men would not be that loyal to a dictator while at the same time sincerely contemplating the well-being of the masses. Most men would either be for the dictator because of the benefits they enjoy or be a counter force like Akubor. But Warunga does not support the dictator and neither does he work against him, even though he recognizes that Ntibantunganyah is evil:

Ever since I started working, my movements have been restricted. My life is an embodiment of sadness. My perceived loyalty is what saves me; but I hate this job, Chief Kajolah. I hate this pretence because I am unreasonably compelled to do things, which I naturally detest. Distress is my companion, sorrow follows me everywhere I go and I feel that I am crawling on the edge of a fragile mist. The mist itself is burdened; it laments at my foolishness (Omobowale 2004, 53).

The balance in the comportment and character Omobowale weaves into Warunga is perhaps a literary technique that might not readily be found in reality. These virtues would hardly combine in one man—understanding different individuals’ peculiar circumstances and empathizing with each one alike, not minding the personal inconveniences this disposition attracts.

Also, the kind of revolution that removes Ntibantunganyah from power is utopian. How many dictators in the real world have committed suicide? How many coups have been realized such that the target(s) and those doing the targeting get destroyed in the process? This does not align with the political realities of Africa.

Every author writes with a mission in mind. A writer’s vision is a comprehensive sense of the objectives of his writings—that is, where he is going and where he wishes to take his readers/viewers. From the crafting of the play, especially in the event that destroys Ntibantunganyah and his army, Omobowale offers an intrinsic socialist vision with a Christian pacifist temper, perhaps because of his religious inclinations, as he seems to re-enact the biblical belief that God fights for the cause of his oppressed people. They are, therefore, to avoid all acts of violence like Warunga, and the wicked will be destroyed by his wicked acts through divine retribution. The refractionist dimension employed by Omobowale, by not reflecting the more common trend of plotting and executing coups but rather creating an ideal one that rids society of all coup plotters, strikes a similarity here too with Ngugi and Mugo’s (1976) handling of *The Trial of Dedan Kimathi*. They do not represent history but recreate it, unlike Ebrahim Hussein (1970) who, like a historian, re-dramatizes Tanzanian history in *Kinjeketile*.

## Conclusion

Generally, African literature is largely art for life’s sake. It often has a functional dimension, a need to address pressing realities. This commitment is mostly seen in the works of African creative writers like Chinua Achebe, Wole Soyinka, Niyi Osundare, Dennis Brutus, Meja Mwangi, and Ngugi wa Thiong’o. Likewise, Omobowale’s play primarily serves an educational purpose; it introduces budding medical practitioners and others to the complex ethics of the profession in a practical way and how the discipline of literature and medicine can enhance a doctor’s proficiency and professionalism (Carson 1994; Lancaster et al. 2000; Kirklin 2001). As Kenneth C. Calman notes, literature makes clinicians

better able to consider human emotions and the response of the patient to disease. It helps them to care. [...] [It] can illuminate aspects of life and make us think in a different way about a particular human emotion or predicament. In doing so, it challenges our existing beliefs and assumptions. [...] [L]iterature may be especially helpful in opening up discussion on ethical issues. [...] The use of literature can provide examples for discussion, debate, and dissention; it allows students and practitioners to test their own beliefs and value systems against others, and it encourages openness and a justification for a certain position—or, for a change of view (Calman 1997, 1620–1621).

## Footnotes

1 Both spellings—“Akampehi” and “Acampehi”—appear in the text, and they refer to the same place (i.e., the name of Chief Kajolah’s community), although the spelling “Akampehi” predominates. “Acampehi” appears only once, in the portion quoted here, and might be a typo from the publishers.
